# Sodium Hypochlorite Reduces Postoperative Discomfort and Painful Early Failure after Carious Exposure and Direct Pulp Capping—Initial Findings of a Randomized Controlled Trial

**DOI:** 10.3390/jcm9082408

**Published:** 2020-07-28

**Authors:** Nidambur Vasudev Ballal, Henry F. Duncan, Namith Rai, Prateek Jalan, Matthias Zehnder

**Affiliations:** 1 Department of Conservative Dentistry & Endodontics, Manipal College of Dental Sciences, Manipal, Manipal Academy of Higher Education, Manipal 576104, Karnataka, India; drballal@yahoo.com (N.V.B.); drnamithrai@gmail.com (N.R.); pjalan41@gmail.com (P.J.); 2 Dublin Dental University Hospital, Trinity College Dublin, University of Dublin, D02 F859 Dublin, Ireland; hal.duncan@dental.tcd.ie; 3 Division of Endodontology, Clinic of Conservative and Preventive Dentistry, University of Zürich, 8032 Zürich, Switzerland

**Keywords:** vital pulp treatment, NaOCl, disinfection, postoperative pain, NRS-11, caries, pulpitis

## Abstract

In this randomized controlled single-center clinical trial on 96 adult patients with parallel experimental groups (*n* = 48), the effects of washing a dentin/pulp wound in non-symptomatic teeth with extremely deep caries and pulpal exposure were compared between a 2.5% sodium hypochlorite (NaOCl) solution and a chemically-inert physiological saline control solution. After the allocated wound lavage, the pulps were capped with a mineral trioxide aggregate, covered by a glass-ionomer/resin liner, and the teeth were immediately restored with a resin-bonded-composite. In this first report, the early events were analyzed: postoperative discomfort (on an NRS-11 scale) at day-3 and -7 after intervention, and the occurrence of unbearable pain causing patients to contact the principal investigator to perform a root canal treatment (pulpectomy) during the first three months. The NaOCl solution caused a highly significant reduction in post-operative discomfort (*p* = 0.0010 day 3; *p* = 0.0007 day 7) and early painful failures (*p* = 0.0008) compared with the control. These novel findings highlight the importance of infection control in teeth with extremely deep carious lesions. Based on these observations, the use of an NaOCl solution to wash the exposed dentin/pulp wound in the vital pulp treatment is highly recommended in order to reduce pain and early failure.

## 1. Introduction

The maintenance of pulp vitality in teeth affected by deep caries is controversial, with restorative dentists and endodontists offering diverging opinions [1,2,3]. Although it is agreed that “minimally invasive” management strategies are preferable, the definition of “minimal” is a source of debate. Systematic reviews analyzing approaches to vital pulp treatment (VPT) have highlighted that primary studies focus on the invasiveness of the operative procedure, i.e., indirect or direct pulp capping versus partial or full pulpotomy, as well as the type of capping material that is placed [4,5]. The outcome measure is invariably the survival, rather than the health of the pulp. Notably, other patient-related outcomes have not gained the same attention [6]. A key patient-centered outcome measure after VPT is postoperative pain, and the subsequent need to see a dentist on an emergency visit to have root canal treatment performed [7]. A randomized controlled trial reported unbearable postoperative pain after VPT procedures to be common, occurring in 7–8% of cases without pulp exposure and 51–63% of cases with pulpal exposure [8], however, other randomized clinical trials have shown considerably less postoperative pain, with severe pain accounting for less than 10% of pulp capping cases [9]. These results highlight that although unbearable pain is possible after management of carious-exposure with VPT, it is not clear how commonly it occurs; however, it does appear to be more likely than after more invasive treatment strategies such as a full pulpectomy [10].

As caries is a biofilm-induced disease [11], treatments aim to arrest lesion progress. Histologically, as the carious infiltration of the dental hard tissues progresses through the dentin towards the pulp, inflammatory reactions in the pulp tissue increase concomitantly [12,13], which rationalizes the focus on the invasiveness of tissue removal during VPT. However, there are other and perhaps more efficient ways to disinfect the dentin/pulp wound. Instead of removing these tissues physically, an alternative and less invasive strategy is to chemically debride the wound. This strategy, i.e., wound lavage, has gained little attention in conservative dentistry, and no randomized trials with an adequate number of patients could be identified [5]. This is surprising, as wound disinfection is a core principle in interventional medicine [14]. However, despite the apparent importance of this procedure, published data on the advantages/disadvantages of individual solutions are sparse, even in a non-dental context [15]. A reason for this may be that wound cleansing studies date back to a time before the dawn of evidence-based medicine. Notably, a 0.5% sodium hypochlorite (NaOCl) solution was identified as the ideal antiseptic to cleanse open wounds during the Great War [16]. In theory, an NaOCl solution would be ideal to wash the dentin/pulp wound in caries-affected teeth, because NaOCl has unique features, including a high efficacy against biofilms combined with semi selective tissue-dissolving properties on necrotic rather than vital soft tissue [17]. There appears to be a trend in recent VPT trials to use NaOCl solutions for this purpose, without the clinical data to support this [18]. On the other hand, while NaOCl has its clear benefits, it may also cause pain because of its caustic potential [19].

This is a first communication from an ongoing randomized trial on the impact of wound lavage on the outcome of direct pulp capping in carious exposures. Here, we report on discomfort after the intervention and from early painful failures, defined as being within the first three months, when 2.5% NaOCl or physiological saline were used to wash the pulp after exposure. The research question asked whether cleaning the pulp wound using an inert sterile solution (physiological saline) resulted in reduced early postoperative pain levels along with higher early treatment failures, defined as an emergency visit to have a pulpectomy (root canal treatment) performed within 3 months after the intervention, compared with a lavage using 2.5% NaOCl.

## 2. Materials and Methods

### 2.1. Study Design

This was a randomized controlled single-center clinical trial on adult patients with parallel experimental groups. The trial was carried out in a university and primary care clinic setting. The patients were blinded and so were the operators (see below). However, because of the specific smell of NaOCl, complete blinding of the operators may not have been possible.

### 2.2. Ethics

The current study protocol was approved by the institutional review board (IEC 881/2018) and registered in the Clinical Trial Registry of India (CTRI/2019/01/017167). It was performed according to the declaration of Helsinki, and kept within the CONSORT guidelines. Other than the randomized treatment step, this study plan did not alter the individual treatment plans in any manner.

### 2.3. Power Analysis

This report was written because there were unexpected early findings that the authors deemed important to share. The sample size analysis for this study was based on the primary outcome measure, which was pulp survival (clinical and radiographic) one year after intervention. Previous prospective clinical comparative trials of greater than one-year in duration have demonstrated that caries exposure, rinsing with NaOCl, and subsequent pulp capping have reported a 90% success rate [18,20,21]. Other carious pulp capping studies using saline [8] or hydrogen peroxide [22] as a pupal rinse have reported lower success of 31% and 55%, respectively. These figures may be attributable to other variables such as the capping material. For the sample size calculation, we assumed that 65% was the appropriate success rate for carious pulp capping at one year rinsed with saline, while 90% represents the success of the NaOCl group. This represents a clinically important difference of 25% on which to base the power calculation. Therefore, a sample size of *n* = 48 per group (96 in total) would achieve 80% power with a type I error of 0.05, type II error of 0.2, and accounting for a 25% drop out. Secondary outcomes for the trial, including postoperative pain and early painful failure, were not subjected to a separate power calculation.

### 2.4. Patient Recruitment

The recruitment phase for this study lasted from January 2019 to February 2020. Adult patients (18 years and older) seeking routine conservative dental care at the Department of Conservative Dentistry and Endodontics were screened for deep carious lesions by three operative dentists involved in this trial (N.V.B., N.R., and P.J.).

### 2.5. Inclusion Criteria

Systemically healthy individuals presenting without complaints of dental pain and with a singular “extremely deep carious lesion” in a periodontally healthy tooth, responding within normal limits to the cold test (Endo-Frost, Coltène, Altstätten, Switzerland) and not showing any rarefaction on the intra oral periapical radiograph, were asked to participate. Teeth displaying an exaggerated or lingering response to cold testing, were excluded from the study. The identification of the study teeth was performed in the course of the normal screening process for new patients clinically and radiographically, according to the criteria set by the European Society of Endodontology [3]. All teeth had a pulpal diagnosis of “reversible pulpitis”, and an apical diagnosis of “normal apical tissues” [23]. Extremely deep caries was defined as “Caries penetrating the entire thickness of the dentin, radiographically detectable when located on an inter-proximal or occlusal surface. Pulp exposure is unavoidable during operative treatment”. These lesions invariably resulted in pulp exposure upon complete caries excavation. Potential participants in this study were handed the patient information sheet (PIS), and were instructed to read it thoroughly. After a “cooling off period”, potential participants were informed that if they were not willing to participate, their treatment plan would remain unaltered. If they were willing to participate after reading the PIS, an appointment was then given for the filling of the selected extremely deep carious lesion. 

### 2.6. Further Screening before Intervention

For specific pain assessment, here and later in the study, the numeric rating scale (NRS-11) was employed [24]. The examiner asked the patient to quantify his/her maximum pain intensity within the last 24 hours on a scale of 0 to 10. The following anchors were used to describe the rating scale: 0 = no pain/pain free and 10 = worst pain imaginable. A score of more than 3 on the NRS-11 scale was used as the exclusion criterion, as this indicates moderate pain [25], which is associated with irreversible pulpitis [13] and a reduced outcome in VPT procedures [8].

### 2.7. Clinical Procedures and Allocation to Treatment

Treatments were performed by three senior clinicians (N.V.B., N.R., and P.J.) with at least 5 years of individual experience as certified endodontists. They used optical magnification (EyeMag Smart, Carl Zeiss, Oberkochen, Germany) to perform the treatments. The tooth was anaesthetized using 2% lidocaine hydrochloride with epinephrine 1:80,000 (Septodont, Saint-Maur-des-Fosses, France), and was isolated under a rubber dam. The cavity was prepared using a diamond-coated point (Horico Dental, Berlin, Germany) in a contra-angle handpiece under constant water-cooling. Caries was excavated using a sterile rose head round bur (Horico Dental, Germany) and discoid excavator (Aesculap Inc., Center Valley, PA, USA). The caries was completely (non-selectively) removed from all of the parts of the cavity. As expected in extremely deep lesions [3], the pulp was exposed in all of the 96 study teeth during this procedure. After pulp exposure, a cotton pellet soaked in sterile physiological saline (non-buffered, non-pyrogenic, pure, sterile 0.9% NaCl, from Fresenius Kabi, Pune, India) was pressed against the wound for one minute in both groups in order to arrest bleeding. 

To avoid selection bias, the randomization of 2.5% NaOCl (KMC Pharmacy, Manipal, India) or physiological saline solution (Fresenius Kabi) occurred after pulp exposure and the arrest of bleeding. This involved picking a closed envelope containing the instruction to either use NaOCl or saline to wash the dentin/pulp wound by an investigator who was not part of this study. Random sequence generation was performed using a computer-generated number (www.randomizer.org), and the block randomization technique (block size of six) and allocation concealment was achieved using the sequential numbered opaque sealed envelope (SNOSE) technique a with a 1:1 allocation ratio. The exposed pulp was then washed according to the allocated treatment for 30 s using either 2.5% NaOCl or sterile physiological saline, using a cotton wool pellet soaked in the respective solution. The solution was handed to the operator in a sterile glass beaker by a researcher not involved in this study. The pellet was pressed against the pulp wound and the cavity was swabbed gently. Subsequently, the cavity was flushed with saline for 10 s and blotted dry using sterile cotton pellets. A hydraulic calcium silicate cement (Medcem MTA, Weinfelden, Switzerland) was then mixed according to the manufacturer’s instructions and placed on the pulp exposure site using a small ball-ended carrier. Once the Mineral Trioxide Aggregate (MTA, Medcem) had initially set, a resin modified glass ionomer liner (Ionolux, VOCO GmbH, Cuxhaven, Germany) was placed over the pulp capping material. Immediately thereafter, the cavity was etched with phosphoric acid (Eco Etch, Ivoclar Vivadent, Liechtenstein) and bonded using Adper Single Bond 2 (3M ESPE St. Paul, MN, USA). The resin-based-composite (Filtek Z350 XT, 3M ESPE, USA) was then placed incrementally into the cavity and was light cured using an LED lamp (3M ESPE, USA), before the occlusion of the patient was evaluated and corrected if necessary. 

### 2.8. Outcome Assessment

The secondary outcomes assessed in this first report of the clinical trial were pain/discomfort assessed on the NRS-11 scale 3 and 7 days after intervention. The patients were contacted via telephone to investigate their pain levels. Furthermore, early emergency visits because of pain in the study tooth were tabulated during the first 90 days after intervention. These emergencies were defined as unscheduled visits by patients in severe pain (NRS-11 score of >6) who required immediate pain relief [7]. In order to avoid the risk of losing patients from the trial to private practitioners outside the University Clinic, all patients were encouraged verbally and in writing to contact the principal investigator (N.V.B.) first, if symptoms arose.

### 2.9. Data Collection, Monitoring and Statistical Analysis

All patient data collected in the participating center were recorded in a designed template and stored in existing patient systems in accordance with data protection rules. A data sheet was completed for each patient to include sex, age, contact details, clinical diagnosis, and allocated intervention. A unique identifier was provided for each patient, which allowed this information to be anonymized and to separate the patient’s name and contact details from the allocated intervention. Data analysis was carried out with oversight from all of the investigators. Categorical data on patient and tooth-related variables were compared between the groups using Fisher’s exact test (two-tailed). The patient ages were compared using the *t*-test. The NRS-11 scores at day 3 and 7 were compared between the groups using non-parametric statistics (Wilcoxon signed-rank test). The potential inter-dependence between the variables assessed in this study and the main study outcome, i.e., early treatment failure, was assessed by Fisher’s exact test (binary variables) or chi-square test of independence. Statistical analyses were computed using JMP software (SAS, Cary, NC, USA). The level of significance for all of these comparisons was set at 5% (*p* < 0.05).

## 3. Results

**Figure 1:**
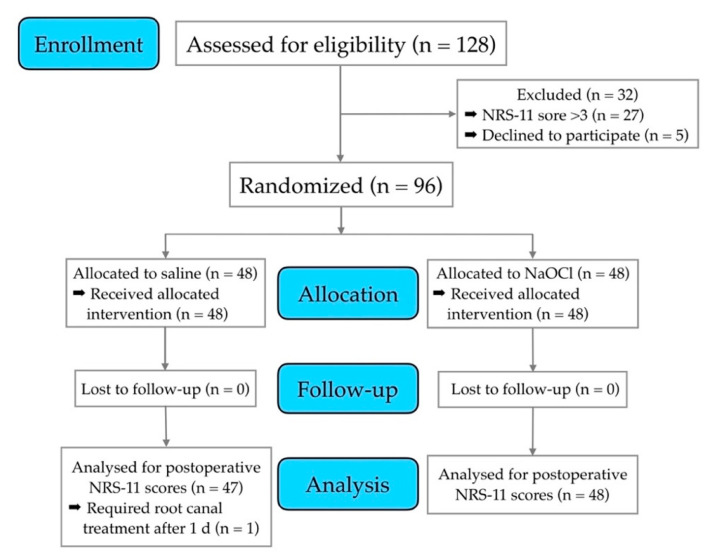
Trial flowchart depicting the participants’ journey through the clinical trial from eligibility screening to analysis. The number of patients that were included and lost with reasons are detailed.

The patient recruitment and all of the pulp capping procedures were completed after 13 months, which included 96 patients requested by the power analysis, who satisfied the inclusion criteria. Approximately 20 to 30 new patients were seen by the investigators per day during their six-day working week, equaling a number of close to ten thousand patients that were pre-screened for the study, resulting in 128 individuals being identified as potential study participants over the study period. Of the 128 potential participants, 27 were excluded, as they had moderate pain (NRS-11 > 3) from the potential study tooth at the day of intervention. Five other individuals who satisfied the inclusion criteria opted out because they could not adhere to the review commitments (Figure 1).

The patient groups allocated to the two treatments under investigation were broadly similar in terms of potential confounding factors (Table 1). The difference in mean age was the only variable that differed significantly (*p* < 0.05) between groups. However, the patient’s age had no significant influence on the occurrence of early treatment failure, and neither did gender, tooth type, cavity class, the jaw the tooth was in, or the operator performing the treatment (*p* > 0.1 for all pairwise tests).

**Figure 2:**
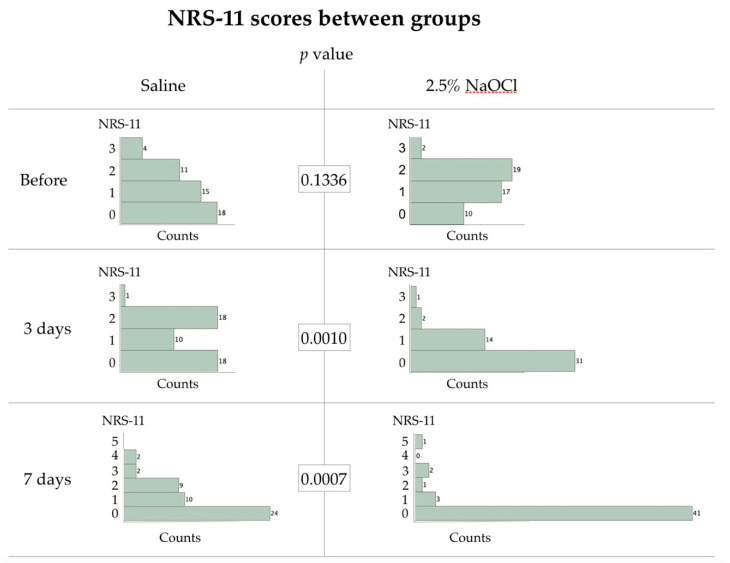
Histograms of numeric rating scale (NRS-11) scores between the allocated treatment groups immediately before and 3 to 7 days after the clinical procedures (complete caries excavation, wound lavage (=randomized step), pulp capping with Mineral Trioxide Aggregate (MTA) and a glass-ionomer/resin liner, and immediate restoration).

The preoperative NRS-11 scores were also similar between groups, with a tendency of the teeth allocated to the 2.5% NaOCl group to cause more preoperative discomfort (Figure 2). This difference, however, was not significant (*p* = 0.1336). On days 3 and 7, the NRS scores became significantly lower in the NaOCl compared with the saline group (*p* = 0.0010 day 3; *p* = 0.0007 day 7; Figure 2). Twelve patients from the saline group reported to the clinic and received root canal treatment due to unbearable pain from the treated tooth within the first 3 months. One of these patients returned on the first day after intervention, with further emergencies treated on days 12, 17, 19, 20, 28, 31, 42, 44 (two patients), 61, and 71. One patient from the NaOCl group presented with unbearable pain on day 17. This difference between groups was also highly significant (*p* = 0.0008). All of the patients who came as emergencies reported spontaneous pain with an intensity of 7–9 on the NRS-11 scale.

The number of failed cases was not large enough to correlate the post-operative pain levels to early treatment failures; however, the one case that reported discomfort at level 5 on day 7 in the NaOCl group (Figure 2) was the same case that appeared on day 17 for emergency root canal treatment. In the saline group, from the two patients who reported level-4 discomfort on day 7, one appeared as an early failure (day 20).

## 4. Discussion

This is the first study to demonstrate a significant effect of the pulp lavage agent on patient-related treatment outcomes. Painful failures of dental procedures on vital teeth are an event that dentists want to avoid, because these emergencies can be hard to manage under local anesthesia [26]. Moreover, painful experiences during or after dental treatments can induce dental anxiety in patients, which, in turn, makes them avoid dental treatments [27]. Washing the dentin/pulp wound with a 2.5% NaOCl solution, which is a simple and inexpensive treatment step that any dentist can manage, significantly reduced both postoperative discomfort and early painful failures in comparison with a control treatment using an inert saline solution. We believe this finding to be both novel and clinically important, and therefore wish to publish these results as rapidly as possible, before the full one-year observation period including the non-painful failures was complete.

In general, pulp exposure should be avoided in asymptomatic cases, with consensus documents suggesting the removal of all deep caries and resulting pulp exposure to be overtreatment [1]. In cases of extremely deep caries, pulp exposure is considered inevitable [3], and for that reason, this cohort of patient were selected for possible inclusion in this study, as indirect pulp capping was not an option. Others may argue that after exposure it is good practice to remove at least a portion of the superficial pulp tissue in a partial or full pulpotomy, techniques which have been shown to yield good clinical results [4]. However, as has been pointed out in recent reviews of the relevant literature, the existing data are heterogeneous and the treatment strategies are so varied that it prevents any conclusions as to which level of intervention is most appropriate to maintain pulp vitality and reduce patient pain [5,28]. In the management of deep/extremely deep caries, each treatment option offers specific advantages and disadvantages, which need to be considered on an individual basis [29]. The current results, however, strongly suggest that disinfection may be more important than the actual volume of pulp tissue removal in avoiding pain and preventing the early failure of the procedure.

It is theoretically possible that NaOCl application to the exposed pulp tissue resulted in the cytotoxicity of neurogenic sensors, which could account for the reduction in evident pain compared with saline lavage in this study. NaOCl exposure to vital tissue in vivo has been linked with neurological damage after extrusion from the root canal into the alveolar bone [30]. Similarly, NaOCl has also been shown to be cytotoxic in vitro when in contact with dental pulpal cell populations, particularly in higher concentrations of 6% [31], which is higher than the 2.5% employed in this study. Cytotoxicity was evident after several hours of incubation, and not within the 30 s contact time employed in this study.

Although this study followed a double-blind design, confounding by guessing the treatment solution by its smell cannot be unequivocally excluded. In future studies on this topic, the operators should wear respirators or take other extra measures to exclude bias from smelling the agent they apply. The randomization led to two fairly uniform groups (Table 1). No factor other than the randomized treatment was associated with the outcomes under investigation. Although statistically significant, an age difference that occurred after the randomization process is unlikely to be clinically important, as several recent studies have not shown patients’ age to be a predictably significant factor in the success of pulp capping in mature teeth [32,33]. A further potential issue with this study relates to pulp exposure and the later arrest of hemorrhage being achieved in every case allocated to the treatment. In any clinical trials, this is unusual, but can be explained by the large number of patients presenting with deep and extremely deep caries at the university clinic. Unfortunately, the prevalence of caries in India is high [34], while there remains considerable value for patients in attending respected dental colleges. Close to ten thousand patients were screened at the department of Conservative Dentistry and Endodontics during the recruitment period for this study (13 months), which may explain how this well-defined group of cases could be identified in this relatively short period of time. Furthermore, patients with pain prior to treatment were excluded. Pain associated with deep caries is a predictor for advanced inflammation and irreversible pulpitis as defined histologically [13]. Therefore, despite the fact that all the teeth under investigation had caries close to the pulp, they were asymptomatic and responded within normal limits to pulp sensibility testing. Therefore, they were likely to be no more than reversibly inflamed, which explains why the bleeding from the pulp space could be stopped with relative ease [35].

The pain assessment tool used in this study is both validated and reliable [25]. Although new online and other established pain intensity scales are available, including the visual analogue scale (VAS) or verbal rating scale (VRS), the NRS scale is considered a practical and reliable index with a good track record [36], and is readily applicable to dentistry [37]. The response rate regarding this outcome was 100% (discounting the one patient who appeared after one day as an emergency; Figure 1). The result of immediate postoperative pain/discomfort reduction observed in this investigation was not expected, as we had hypothesized that NaOCl solutions can have inflammatory effects on vital tissues [19]. On the other hand, on clean-cut pulp wounds, the type of material or medicament that is in contact with the wound surface may not exert a significant role in terms of postoperative pain [38]. The finding that there was actually less postoperative pain when an NaOCl solution was used for wound lavage rather than saline would suggest that early painful events are likely to be due to infection rather than chemically induced inflammation, although we cannot speculate if the results would have been similar if a stronger NaOCl (e.g., 5%) had been used in this study.

For cases presenting with unbearable pain requiring root canal treatment (pulpectomy), an open approach was chosen, i.e., patients were not recalled at the 3-months interval. A 3-month recall visit would have missed the dynamic presentation of this early failure group, which by definition is unscheduled.

Overall, these results strongly support the use of a 2.5% NaOCl solution for wound lavage after carious exposures. Most recent studies on VPT used NaOCl solutions for this purpose, while older investigations did not [5]. This trend was likely propagated by endodontic researchers clinically working with microscopes and knowing about the unique features of NaOCl in terms of necrotic tissue and biofilm dissolution [3]. However, hitherto, the recommendation to use NaOCl solutions for the current purpose has not been substantiated by any data. Interestingly, dentin wound disinfectants used to be popular, especially in the form of a dentin washing solution containing benzalkonium chloride, a quaternary ammonium compound (Tubulicid, Dental Therapeutics, Nacka, Sweden). Whether such solutions are helpful in washing dentin/pulp wounds under carious exposures, however, remains to be investigated. NaOCl, which happens to be the core component of a product for chemical caries removal (Carisolv, MediTeam Dental, Göteborgsägen, Sweden), uniquely debrides necrotic soft tissues and biofilm. Furthermore, if followed by a rinse with an inert solution, it does not interfere with dentin bonding. To the contrary, by dissolving an organic material that may have been exposed in the acid environment of the carious lesions, it can improve the bond strengths and hence may just be the perfect agent for the current purpose [39]. Nevertheless, future studies could compare different antimicrobial agents for the lavage of the dentin/pulp wound. Moreover, the ideal concentration of NaOCl solutions could be defined. 

## 5. Conclusions

This randomized trial showed that washing the dentin/pulp wound with a 2.5% NaOCl solution in non-symptomatic teeth with extremely deep caries caused a highly significant reduction in post-operative discomfort and early painful failures compared with a control treatment using an inert saline solution. These findings highlight the importance of infection control in such teeth. They also indicate that, despite its potential caustic effects on vital tissues, the NaOCl solution under investigation did not induce immediate postoperative discomfort in this application. The use of an NaOCl solution in vital pulp treatment should thus be highly recommended based on these findings.

## Figures and Tables

**Table 1:** Independent variables according to allocated treatment.

	Saline	2.5% NaOCl	*p* Value
Patient age (y)	38 ± 12	30 ± 12	0.0011 *
Patient gender (f/m)	32/16	23/25	0.0981
Tooth type (pm/m)	14/34	12/36	0.8187
Cavity class (Black I/II)	4/44	7/41	0.5235
Jaw (maxilla/mandible)	22/26	24/24	0.8383
Operator (N.V.B/N.R./P.J.)	19/13/16	16/17/15	0.6627

y = years; f = female, m = male; pm = premolar; m = molar; * significant difference (*t*-test) between groups.
